# The Sequelæ of Enteric Fever—III

**Published:** 1908-06-20

**Authors:** 


### THE SEQUELÆ OF ENTERIC FEVER—III.

### Gall-stones.

In not a few instances cultures made from the interior of gall-stones have yielded the bacillus typhosus many years after the occurrence of typhoid fever. There is, indeed, a general consensus of opinion that typhoid fever predisposes to biliary calculi in later life. The gall-stones do not reveal themselves for many years as a rule, and the typhoid fever may have been quite forgotten; but the fact that the bacilli can persist in them so long is not surprising when, as in the cases of pyelitis and periostitis discussed in our last article, they are known to be latent, but alive, in other places for periods varying from ten to forty-six years.

The relation of micro-organisms to gall-stones is very interesting. Evidence is accumulating to show that biliary calculi never arise except when there has been a preceding microbial catarrh of the gall bladder. Some very instructive experiments in this connection have been made upon dogs: human gall-stones having been obtained and sterilised, they were introduced with all aseptic precautions into the gall-bladders of dogs; after a variable number of weeks the animals were again operated upon and the gall-bladders examined; the human gall-stones had disappeared. A second series of experiments was then carried out, all the conditions being the same except that, instead of the gall-stones being put into the gall-bladders aseptically, a measured quantity of a bacterial culture was introduced along with each calculus; the number of micro-organisms was regulated in such a way that the inflammation in the gall-bladder should stop short of empyema—the result being noted after a few weeks as before; the gall-stones had increased in size instead of disappearing as in the aseptic cases. In other words, according as there were or were not bacteria in the gall bladder, the gall-stones increased in size or diminished and disappeared. The significance of these experiments is very great. It is probable that typhoid bacilli infect the gall-bladder and cause a moderate cholecystitis in a good many cases in which the mischief stops short of pus formation; the cholecystitis leads to the deposition of calcium salts of bile pigments, matted together with mucus, to form nuclei around which cholesterin is to be deposited later, the final result being a typical gall-stone or several of them. Inhibition of the growth of the microbes in the gall-bladder should be effected by giving urotropine after the typhoid fever, as we have already described, and a proper flow of bile should be ensured by seeing that the patient does not restrict the daily intake of fluid too much. It is probable that in human beings, as in dogs, gall-stones will dwindle and disappear if bacteria in the gall-bladder can be removed.

### Impairment of Hearing.

In a certain proportion of cases of typhoid fever there is a considerable degree of deafness during the pyrexial period. The patients are often so lethargic that they do not complain of this spontaneously; it is probable that the symptom is more common than at first sight might appear. Fortunately the impairment of hearing is transitory and free from danger; it is perhaps a good thing in disguise, for it saves the patient from being worried by the various noises round about him. In a few cases, however, the partial deafness is not so transitory. It may be noticed for the first time after the patient has begun to get better, and it may persist for months or even for life. It comes, therefore, under the heading of sequelæ. Its pathology is uncertain; probably there are more causes for it than one. It might be thought to be due to tonsillitis, with the extension of the inflammation to the orifices of the Eustachian tubes; but this is not the cause in every case, for tonsillitis is not a very common thing in enteric fever. In a few cases it may be due to a degenerative lesion of the auditory nerve; but this again cannot be the cause in most cases, for even when the deafness has been almost complete for a time, considerable recovery is the rule. A third explanation seems to be the most probable—namely, that it is due to non-suppurative inflammation of the periosteum of the petrous bone. Such an inflammation would be comparable to the nodes, and like them it might resolve completely, leave behind it permanent thickening, or go on to suppuration. We find, in correspondence with this, cases in which hearing becomes completely restored, cases in which hearing remains to a greater or less extent defective, and cases in which purulent otitis media with chronic otorrhœa results.

In the same class with those in whom hearing is simply impaired would come those unfortunate persons in whom the chronic bony or periosteal thickening leads to irritation of the middle ear or auditory nerve, with consequent subjective sensations of tinnitus, a condition of things which it is exceedingly troublesome to treat. Potassium iodide, in virtue of its reputed powers of absorbing the results of inflammation, might possibly do good if it were given early.

### Ulceration of the Larynx.

Another sequela of typhoid fever, though fortunately not a common one, is ulceration of the larynx with hoarseness, and pain on swallowing. Unless the possibility is borne in mind it may be thought that the patient's illness has either lit up some latent tubercle, or else has caused a dormant syphilis to become become active in the throat. The lesion is essentially comparable, from a pathological standpoint, with the abscesses, the periosteal nodes, and the affections of hearing that we have discussed, for it is due to a perichondritis. The perichondrium of the arytenoid cartilages becomes inflamed; in the milder cases the trouble stops short of pus-formation, but for the time being, and perhaps for many weeks or months, it interferes with the free movement of these cartilages, and therefore with that of the vocal cords. When actual ulceration occurs, it is due to pus forming beneath the perichondrium and then bursting through it, leaving a single deep ulcer at the posterior end of each vocal cord with the bared arytenoid cartilage in the bottom of the ulcer. Multiple ulcers are unusual. Healing is a slow process, much impeded by the exfoliation of part of the necrosed cartilages, but cure is possible, the prognosis being infinitely better than it is, for example, in tuberculous ulceration of the larynx.

